# Bacterial Contamination of Ultrafiltration Installation Applied to Carwash Wastewater Treatment

**DOI:** 10.3390/membranes15030071

**Published:** 2025-03-01

**Authors:** Piotr Woźniak, Marek Gryta

**Affiliations:** Faculty of Chemical Technology and Engineering, West Pomeranian University of Technology in Szczecin, ul. Pułaskiego 10, 70-322 Szczecin, Poland; piotr.wozniak@zut.edu.pl

**Keywords:** ultrafiltration, biofouling, chemical washing, car wash

## Abstract

An ultrafiltration (UF) installation was used to separate the actual wastewater from a car wash. Following these studies, the plant was washed several times; however, severe membrane fouling was observed during the filtration of sterile deionised (DI) water. As a result, the permeate flux decreased by more than 50% after 5 h of the UF process. The source of the fouling was the release of deposits, particularly bacteria, from the surfaces of plant elements such as pipes and pumps. The paper presents the effectiveness of biofilm removal from the surface of the equipment during a cyclically repeated washing process. Chemical washing was carried out using acid solutions and alkaline cleaning solutions containing NaOH (pH = 11.5–12). After installation cleaning, the filtration tests were carried out using DI water as a feed. It was determined how biofouling, which develops under these conditions, reduces permeate flux. Despite 3 h of installation washing, there was a 50% reduction in flux after 10 h of UF. Repeating the installation wash (4 h) resulted in a similar decrease in flux after 4 days of UF. Stabilisation of the flux at a level of 500 LMH was achieved after an additional 5 h of washing, including application of hot (323–333 K) alkaline cleaning solutions. The number of bacteria in the biofilm collected from the surface of the membranes, the pump inlet and the surface of the polyvinyl chloride (PVC) hoses forming the pipeline was also investigated. Despite repeated chemical cleaning, the number of bacteria on the pump and hose surfaces was 50–100 CFU/cm^2^. Studies were carried out to determine which bacterial species survived the chemical cleaning of the installation. Gram-positive and Gram-negative bacteria were determined, and taxonomic characteristics of the isolated bacteria were identified.

## 1. Introduction

Huge amounts of water, estimated in millions of cubic meters, are used annually to wash cars [1,2]. For this reason, technologies are being developed using a variety of traditional treatment techniques to recover wash water from the wastewater generated by car washes [3,4,5,6]. The use of membrane separation also offers such opportunities [7,8,9]. One limitation is that wastewater causes significant membrane fouling, which is reduced by cyclic module cleaning [10,11,12] or application of self-cleaning membranes [13]. Membrane fouling can be mitigated by using the feed pre-treatment, which is often combined with flotation, coagulation and filtration through different beds [8,14,15,16]. However, the use of multi-stage processes increases costs, and such methods will not be applicable in small carwashes. In this case, an effective solution to reduce fouling has been developed by applying alkaline agents used in carwashes [17].

The size of the carwash market, valued at USD 1.72 billion (2024), is increasing every year [18]. In addition to large automatic carwashes, small touchless washes are also popular. The effluents generated during car washing are collected in a settling tank (removal of coarse particles and sand), from which they flow through an oil separator into sewage systems. The touchless car washes are fed with tap water, the price of which in Poland is around 5–7 euros/m^3^ including disposal. This low price means that water reuse technologies must be simple and cheap. This can be achieved with a UF system supported by car wash equipment such as dosing pumps, computer controls and cleaning agents used in car washing [11,17].

In addition to the pollutants removed from cars, carwash wastewater contains numerous bacteria, including some that are resistant to antibiotics [19]. Membrane separation, such as ultrafiltration (UF), removes them from the water, but bacteria can proliferate intensively in the membrane installation [12,17]. As a result, bacteria can accumulate on the membrane surface (biofouling), which significantly affects the operation of membrane modules [20,21,22]. The type of microbial species and the membrane properties play an essential role in biofilm formation and structure [23]. Microorganisms usually produce extracellular polymeric substances (EPS), which are responsible for the growth and adhesion of microorganisms on the surface. EPS is mainly composed of polysaccharide and protein [24,25]. The composition of the biofilm depends on the type of bacteria forming it [26]. Caustic cleaning involves the hydrolysis and solubilisation of foulants such as proteins and saccharides [25]. The development of biofouling during separation of car wash wastewater was reduced by daily washing of the UF installation with an alkaline cleaning agent [17].

The biofilm forms a barrier between the membrane surface and the feed solution, increasing the mass transfer resistance and leading to a decrease in the permeate flux and feed separation. Therefore, periodic cleaning of membranes in industrial plants is necessary. Typically, physical methods such as backwashing and turbulent mixing and/or chemical washing are used [20,25,26]. In general, cleaning solutions contain a base, oxidant, surfactant and chelating agent. The primary role of the caustic wash step is to remove proteins and carbohydrates. In turn, the role of the acid step is to remove mineral scale, remove traces of alkaline product and provide bacteriostatic conditions [27]. The use of hot acid and alkaline solutions can also effectively remove bacteria in hard-to-reach areas of the system [27,28]. In the case of biofilms formed during wastewater treatment, NaOH solutions allowed recovery of more than 80% of membrane performance [24,26]. The use of professional detergents containing surfactants and chelating agents (ethylenediaminetetraacetic acid—EDTA) in addition to NaOH significantly increases the efficiency of plant cleaning [27]. However, biofouling cannot usually be completely eliminated by periodic pre-treatment or post-treatment with biocides and other chemicals [16]. In addition, chemical cleaning agents (e.g., HCl, NaOH and NaClO) can intensify biofouling during the UF process [24]. As a result, more resistant microorganisms exposed to harsh/unfavourable conditions will produce biofilm, causing a reduction in flux. Working conditions can be further exacerbated by the fact that touchless carwashes do not operate continuously, which can facilitate the development of biofouling. It is therefore important to develop a cleaning procedure for the UF system, especially after prolonged downtime.

Some microbial species, such as *Bacillus cereus*, show significant resistance to cleaners [29]. These bacteria show strong adhesion to stainless steel, a material widely used in membrane installations. Due to spore formation, *B. cereus* can survive high-temperature pasteurisation processes, if appropriately conducted [30]. In addition, these bacteria can survive and proliferate at pH 5–10 and are resistant to high salt concentrations [31,32]. The food industry sterilises equipment with steam, which cannot be used in polymeric membranes due to its excessive heat (over 400 K).

The use of clean-in-place (CIP) systems in industrial plants reduces the development of fouling/biofouling, allowing the membrane system to operate efficiently [12,21,27]. For small carwashes, this solution has two main limitations: (a) it is too expensive, and (b) the cleaning agents used are too aggressive for car paints. For this reason, car wash chemicals have been proposed to clean UF membranes and reduce fouling [11,17,33]. Furthermore, only the influence of feed components on membrane fouling is usually studied [34]. Meanwhile, biofilms form on the entire surface of the installation, such as pipes, and their influence on biofouling has not been studied. This may be of significant importance in the case of manual car washes, where the periods of standstill favour the growth of bacteria in the installation. 

Most papers on biofouling present studies on the formation of the initial biofouling profile using pre-sterilised laboratory equipment and new membrane samples [20,24]. This differs from conditions in industrial plants, where usually only a new membrane module can be installed. On the other hand, the system of tanks, piping, valves and pumps always creates hard-to-reach areas where bacteria can grow, despite intensive cleaning of the plant. Probably for this reason, after repeated washing of an UF plant previously used to separate the actual car wash wastewater, membrane fouling still occurred during filtration of sterile DI water. The current work presented here investigated the reasons for this and whether the use of chemical cleaning of the UF installation could eliminate the formation of biofilm on the membrane surface.

## 2. Materials and Methods

### 2.1. UF Installation

**Figure 1:**
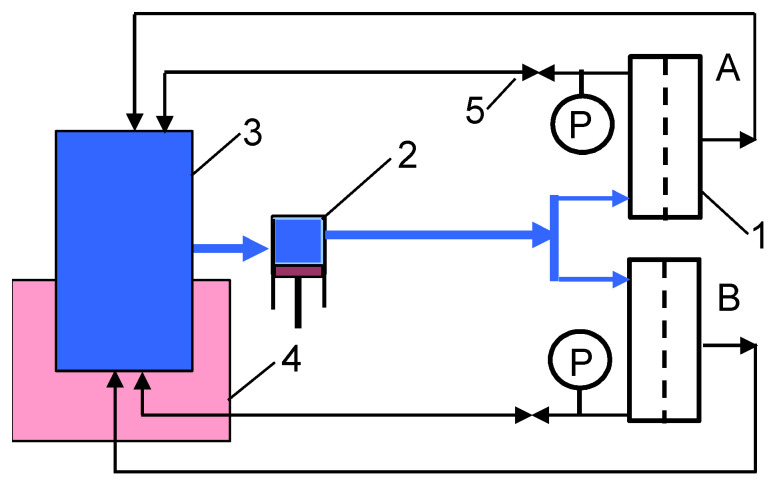
Experimental set-up of the UF installation with two plate-and-frame membrane modules (A and B). 1—module, 2—pump, 3—feed tank, 4—thermostat, 5—valve and P—manometer.

The experimental setup is presented in Figure 1. The installation was equipped with a 3CP Stainless Steel Plunger Pump model 3CP1221 (CAT PUMPS, Hampshire, England). This type of pump is commonly used in manual car washes. The pump fed laboratory cross-flow filtration cells connected in parallel with an identical experimental design. This allowed membrane fouling investigations to be carried out in two modules under equivalent conditions. The module design is presented in [35]. The channels in the modules (dimensions 40 × 60 × 0.7 mm) were filled with polypropylene (PP) net spacers (orthogonal, 20 mesh). The modules were made of acid-resistant steel (AISI 316), from which the valves and pressure gauges were also made. Polyvinyl chloride (PVC) hoses were used to make a pipeline (total internal area 687 cm^2^) connecting the modules to the pump and glass feed tank. During the test, 2 liters of the liquids were poured into the tank.

The modules were equipped with commercial ultrafiltration polyethersulfone (PES) membranes UE50 (100 kDa) and UE10 (10 kDa) manufactured by TriSep Corporation (Goleta, CA, USA). The membrane working area was 24 cm^2^. The UF tests were performed at transmembrane pressures (TMP) of 0.1 and 0.3 MPa. During membrane washing, the valves (5) were fully open (TMP = 0). The applied feed flow rate was 1 m/s. After passing through the modules, the feed was returned to the feed tank. Using sterile deionised (DI) water (Elix 3, Millipore, MA, USA) as the feed, the UF process was conducted for 5–7 hours per day, after which the plant was flushed with fresh DI water (2L). After an overnight shutdown, the system was rinsed (2 liters of DI water) and filled with a fresh batch of DI water, and filtration resumed. 

### 2.2. Cleaning Solutions

The UF installation used in the present study was washed with cleaning agents applied in industrial membrane systems [26,27]. In addition to acid rinsing, cleaning with alkaline agents such as Ultrasil was also realised [36].

The UF-tested installation (Figure 1) was used for several weeks to separate wastewater collected from a car wash [16]. More than 30 types of bacteria were detected in the applied wastewater [19]. After these experiments, the unit was repeatedly rinsed with DI water, 0.5% H_3_PO_4_ solution (ChemLand, Stargard, Poland) and finally with alkaline agents, the composition of which is shown in Table 1. The commercial agent P3 Ultrasil 11 (SUTURAMED, Szczecin, Poland), recommended for UF membrane cleaning [36,37], was used in a 0.25% solution (pH = 12). NaOH is also contained in the Insect agent (EuroEcol, Łódź, Poland) used for car washing, from which a 0.5% solution (pH = 11.5) was prepared. This agent has been successfully used for the cleaning of membranes that have been contaminated during the separation of real car wash wastewater [33].

The above cleaning agents were also used in the present tests when the UF installation was restarted after a few weeks of standstill. In the experiments carried out, the UF plant was washed with cleaning solutions at 293–295 K. In addition, a long (over 1 h) high-temperature (323–333 K) wash of the plant was carried out, mostly without membranes mounted in the UF modules. Polymer membranes can usually withstand such high temperatures, but module seals can be damaged. For these reasons, membrane module manufacturers generally do not allow such operating conditions. In order to avoid damage to the modules during high-temperature cleaning of the installation, the modules must be disconnected, for example, by using bypasses.

### 2.3. Bacterial Analysis

**Figure 2:**
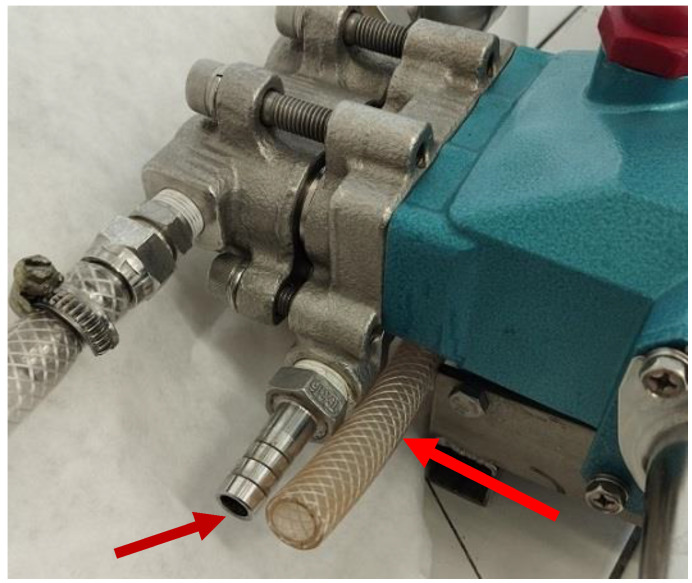
PVC hose disconnected from a 3CP Stainless Steel Plunger Pump to take a sample of the biofilm from the inside of the hose and the inlet port to the pump. Arrows—biofilm collection point.

Biofilm was removed from the membrane surface (1 × 1 cm area) using a sterile swab, which was then immersed in 10 mL of a 0.9% NaCl solution. Biofilm was similarly sampled from the PVC surface of the hose and from the inlet port of the pump (Figure 2). Another study has shown that wiping the surface of steel with a cotton swab can collect almost all bacteria present on the surface [27].

The NaCl solution with the immersed swab was vortexed for 2 min, and 0.3 mL of the resulting bacterial suspension was transferred to Petri dishes. Bacterial cultures were performed on Petri dishes using media manufactured (BioMaxima, Lublin, Poland), in particular non-selective media according to standard methods (plate count agar). All types of cultures were performed in triplicate, and the cell counts were expressed as mean CFU/mL. 

Isolated bacterial colonies were transferred to Columbia LAB-AGAR™ Base non-selective medium (BioMaxima, Lublin, Poland) for further cultivation. The resulting pure bacterial cultures were then placed on a metal targeting plate designed for MALDI-TOF/MS analysis and dried after matrix application. Microbial identification was performed by mass spectrometry using a Bruker MALDI Biotyper® (MBT) instrument (Bruker Daltonik GmbH, Bremen, Germany). Identification of bacterial species was achieved by comparing the mass spectrum of the microorganism under investigation with the IVD MBT mass spectrum reference library.

#### Taxonomic Characteristics of the Isolated Bacteria

Bruker’s MALDI Biotyper has made it possible to identify the bacterial species in wastewater, for which data for taxonomic analysis are available in the National Center for Biotechnology Information database (https://www.ncbi.nlm.nih.gov/, accessed on 15 December 2024). The 16S rRNA gene sequences retrieved from this database (option: NCBI/EZtaxon/Ribosomal Database Project, accessed on 15 December 2024) were used to assess the phylogenetic tree of the bacteria identified on the membrane surface, using the BLAST (blastn) program (http://www.ncbi.nlm.nih.gov/BLAST/, accessed on 15 December 2024). All sequences were aligned using Clustal W, and a maximum likelihood (ML) phylogenetic tree was constructed using the MEGA software package, version 11.

## 3. Results

### 3.1. Restart of UF Installation

The UF installation shown in Figure 1 was used to study the separation of wastewater from a car wash. Studies with wastewater containing 2.8–3.7 × 10^6^ CFU/mL of bacteria [19] were carried out over several weeks. Daily membrane rinsing with 0.5% Insect solution (pH = 11.5) kept the permeate flux stable at 50% of the initial flux value [17]. After the study, the system was washed several times with P3 Ultrasil 11 and Insect solutions (pH = 11–12). The UF installation was then thoroughly rinsed with DI water, the liquids were removed and the filtration cells were opened. The system was not used in this condition for several weeks.

**Figure 3:**
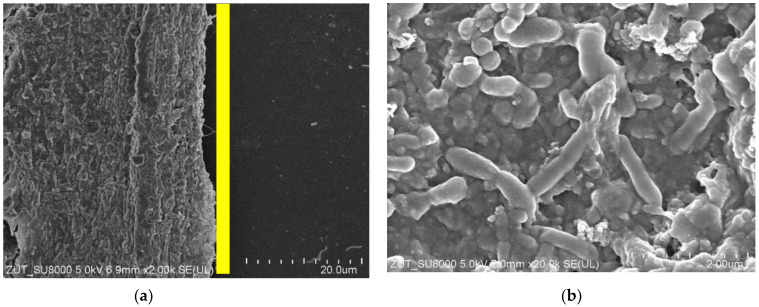
SEM images of UE50 membranes (module A): (**a**) yellow line—seal location in UF module, to its left side active membrane area with deposit; (**b**) enlarged image of deposit.

For the subsequent tests, the UF system (without membrane in the modules) was first rinsed with DI water (1 h). The installations were then washed with phosphoric acid solution (0.5%) for over 1 hour, followed by a 0.5% Insect solution (1 h). After these operations, the installations were rinsed several times with DI water. After the installation was cleaned, the UE50 membranes were installed in the modules, and filtration was carried out with DI water as the feed for 5 h at TMP = 0.3 MPa. During this time, a rapid decrease in permeate flux was observed, with the value decreasing from 1850 to 767 LMH. When the modules were opened, it was observed that the colour of the membranes changed from white to brown, which indicated a significant fouling phenomenon. Moreover, SEM examination showed that the deposits formed on the membrane surface contained numerous bacteria (Figure 3). Although acid and alkaline wash is considered effective [27,28], in this case it did not remove deposits from the installation surface, the residues of which were detached during DI water filtration and caused membrane fouling. 

### 3.2. Influence of Membrane Permeability

The next step was to thoroughly wipe off the deposits visible inside the UF modules using filter paper soaked in isopropanol, after which the UF plant (without the membranes) was cleaned again. It was washed with P3 Ultrasil 11 solution (pH = 12) for 3 hours and then rinsed several times with DI water. It does not contain bacteria and does not cause their intensive growth. It can therefore be assumed that the deposits shown in Figure 3 are mainly due to the formation of a filter cake from contaminants detached from the surface of the UF installation.

**Figure 4:**
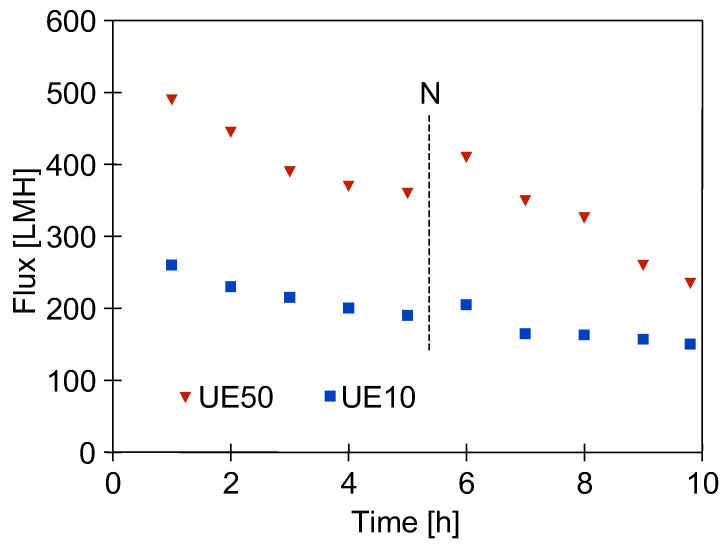
Permeate flux changes during 2 days of DI water filtration tests. Point N–overnight shutdown.

**Figure 5:**
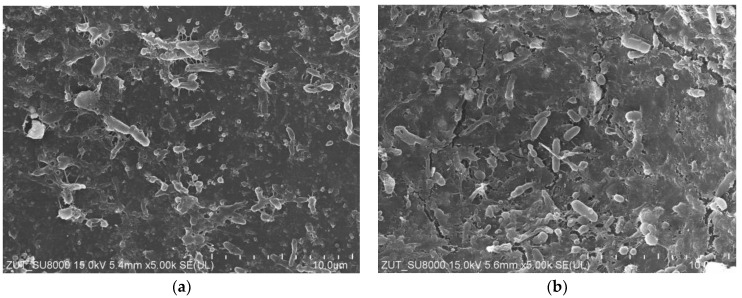
SEM images of deposits on the PES membranes surface after 2 days filtration of DI water. (**a**) Membrane UE10 (module A), (**b**) membrane UE50 (module B).

The formation of the filter cake depends to a large extent on the permeate flux [38]. Therefore, in order to estimate its influence, the UE10 membrane (10 kDa) was installed in module A, and the more permeable UE50 membrane (100 kDa) was installed in module B (Figure 1). Next, the DI water filtration was started with TMP = 0.1 MPa. The filtration process was conducted for 5 h/day, after which the UF units were rinsed twice with DI water. The results of the 2-day tests are shown in Figure 4. Despite several hours of installation washing, there was still a decrease in the permeate flux. It was greater for the more permeable UE50 membrane, which confirms the conclusions presented in the literature [38]. Analysis using the Hermia model indicated that the membrane with MWCO of 100 kDa is more prone to intermediate blocking. SEM studies showed that there was a slightly larger amount of deposits on the surface of this membrane. However, the amount of deposits was much smaller than that formed after the first washing (Figure 5). UE50 membranes captured contaminants from the feed to a greater extent; hence, in the next UF tests, they were used as an indicator of the intensity of detachment of deposits from the surface of the tested UF installation. 

### 3.3. Influence of Extended Cleaning Time

The obtained results indicate that despite the chemical cleaning of the UF plant, which was carried out twice, a complete removal of deposits from its parts was not achieved. Therefore, in the next stage of the research, the period of chemical cleaning of the installation was extended. It was first washed with P3 Ultrasil 11 solution (pH = 12) for 3 h, followed by 0.5% H_3_PO_4_ solution (1 h) and then rinsed several times with DI water. The next day, the installation was washed with 0.5% Insect solution (pH = 11.5) for 1 hour and finally rinsed thoroughly with DI water. Once the cleaning process was complete, the UF modules were opened and thoroughly cleaned with isopropanol.

**Figure 6:**
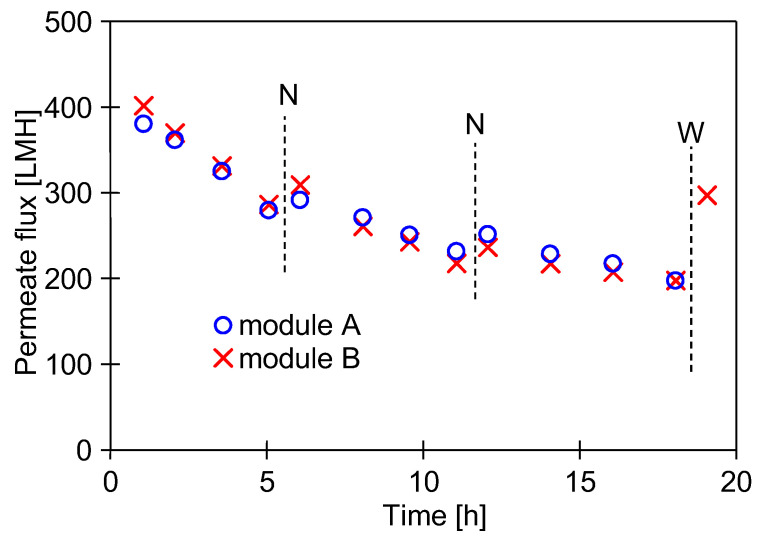
Permeate flux changes during 3 days of DI water filtration tests. Point N–overnight shutdown, W–membrane washing with P3 Ultrasil 11 solution (pH = 12, T = 313–323 K).

After installing the UE50 membranes, DI water filtration was started at TMP = 0.1 MPa. The filtration process was carried out for 3 consecutive days (5–6 h/day), during which the permeate flux decreased from 385 to 230 LMH (Figure 6). Although the daily flux decreases were somewhat smaller than those observed in the previous series (Figure 4, ‘UE50’), they were still significant. It was noted that the colour of the membranes changed from white to light brown, indicating that fouling was still occurring. It follows that repeated washing with NaOH and H_3_PO_4_ solutions did not remove all the deposits from the UF installation surface, the remnants of which were detached and deposited on the membrane surface. This phenomenon occurred mainly on the first day, after which the flux stabilised in the following days. The membrane from module A was collected for SEM studies, and the system with module B was then washed for 1 hour with P3 Ultrasil 11 solution heated to 313–323 K. As a result of this operation, the permeate flux increased only to 295 LMH (Figure 6, point ‘W’). This finding indicates that the deposits formed on the membrane surface are difficult to remove even by hot cleaning agents. This explains why washing the membrane with DI water after the overnight shutdown resulted in only a slight increase in flux (Figure 6, point ‘N’).

**Figure 7:**
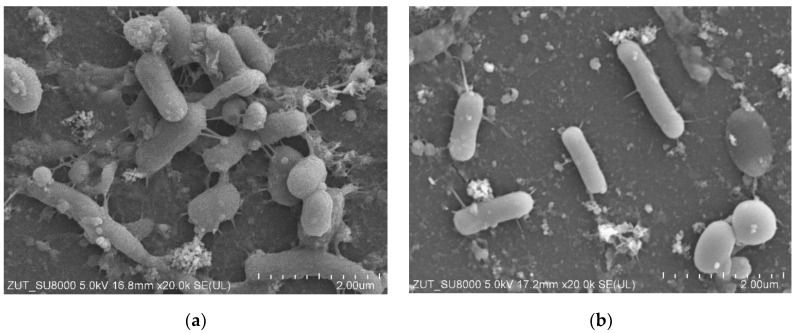
SEM images of deposits on the UE50 membranes surface (**a**) after 3 days filtration of distilled water (module A); (**b**) after membrane washing (module B).

SEM studies confirmed that the reason for the decrease in permeate flux observed during DI water filtration was the formation of deposits on the membrane surface (Figure 7a). It could be seen that the deposits also contained numerous bacteria. However, the amount of deposits was significantly less than that observed after the previous UF unit cleaning (Figure 3 and Figure 5b). This indicates that the use of additional extended cleaning time had positive results.

Washing the fouled membranes for 1 hour with P3 Ultrasil 11 solution (Figure 6, point ‘W’) removed some of the deposits, but the bacteria were still present on the membrane surface (Figure 7b). This result confirms the conclusions of other work that even chemical washing does not completely remove the biofilm [12,16,24]. It has been observed (Figure 7) that rod-shaped bacteria possess a hair-like nanofibre called a pilus or fimbria [39]. Their formation facilitates bacterial attachment to the colonised surface [28,39]. This indicates that the deposit on the membranes was not only formed by the deposition of contaminants detached from the UF installation surface, but also by bacterial growth. The development of biofouling during the separation of car wash effluent was reduced by daily rinsing of the plant with a 0.5% Insect solution [17]. A 5-litre concentrate of this cleaning agent costs 30 euros and is sufficient for approximately 100 rinses of an UF system with one to two modules.

### 3.4. Nutrients and Bacterial Growth

Bacteria need nutrients to grow; therefore, reducing their concentration in the feed will reduce the biofouling tendency in the membrane system [40]. In the case studied, the UF installation was fed with deionised water, which should inhibit bacterial growth. However, as shown in another paper, even in a DI water environment, bacteria can survive by forming a biofilm [41]. In industrial plants, the biofilm is usually formed by mixed species [23]. Such biofilms host several species of bacteria that can capture trace amounts of nutrients, such as Fe by *Pseudomonas aeruginosa* bacteria [42], which supports biofilm formation [39]. For these reasons, the use of DI water as a feed did not eliminate the development of biofouling. The nutrients could be contaminants washed out from the surface of the installation.

SEM-EDX analysis of the deposits on the membrane surface (Figure 7a) showed the presence of small amounts of P, Fe, Mg and Ca, which bacteria can use to grow. Washing the membrane removed some of the deposits from the surface (Figure 7b), and, as a result, the concentrations of these elements decreased (Table 2). However, important components for bacterial growth, such as P and Ca, remained on the membrane surface. Even trace amounts of phosphorus have been found to enhance biofilm development and growth [43]. Chemical cleaning can inactivate bacteria but generally does not completely remove the biofilm and its constituents from the installation surface. As a result, the remaining organic matter and trace elements can be utilised as nutrients for the growth of bacteria that have survived the cleaning process [32].

At the end of the UF test, the results of which are shown in Figure 6, the UF installation was rinsed with DI water and not used for 3 days. The results obtained (Figure 7) showed that despite repeated cleaning, viable bacteria were still present in the UF installation. Therefore, biofilm development can occur during the downtime of the system. Small carwashes may operate with pauses, and it is not expected that preservatives normally used in industrial membrane systems will be used during the pauses.

### 3.5. Influence of High-Temperature Washing

**Figure 8:**
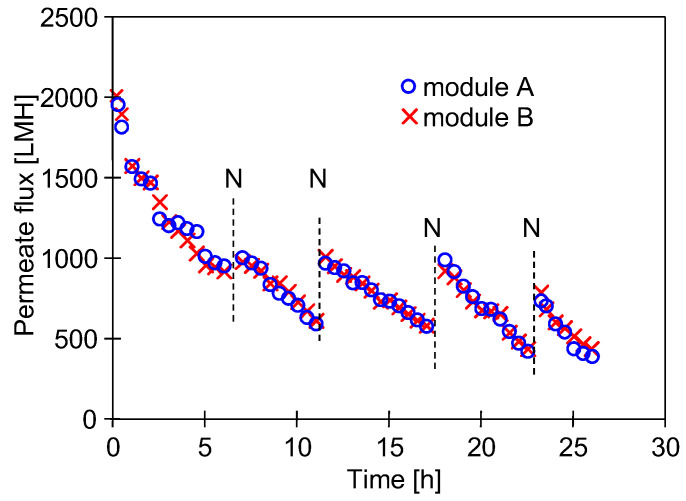
Changes in the permeate flux during filtration of DI water. TMP = 0.3 MPa. Point N—overnight shutdown.

In anticipation of an increase in biofouling, the washing intensity of the UF system has been increased. It has been documented that the use of hot cleaning solutions increases the removal of biofilm [27,28]. Therefore, in the next test, the wash temperature was increased to 333 K, and the subsequent washing of the system (1 h) was carried out with hot solutions of 0.5% H_3_PO_4_ and P3 Ultrasil 11, followed by DI water rinse (5 h, T = 293 K). After washing, the new UE50 membranes were installed, and DI water filtration was performed (6–7 h/day, TMP = 0.3 MPa)—Figure 8.

**Figure 9:**
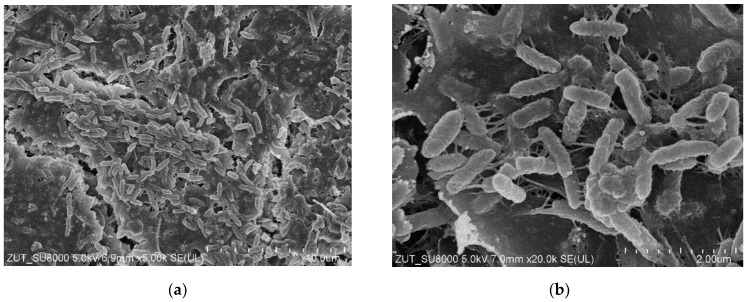
SEM images of UE50 membrane after 5 days filtration of DI water (Figure 8). (**a**) Deposits on the membrane surface; (**b**) Bacteria inside deposits. Sample taken from module A.

To reduce bacterial growth during the overnight shutdown, after the UF process, the installation was additionally washed every day (0.5 h) with 0.5% Insect solution (pH = 11.5). After an overnight shutdown, the permeate flux increased due to membrane decompression (TMP = 0) and membrane flushing with DI water (Figure 8, point ‘N’). The repeatability of the change in permeate flux and its stabilisation at 500 LMH over several series indicates that the high-temperature washing process and daily rinsing of the equipment reduced, but did not eliminate, biofilm growth. SEM studies confirmed that fouling was the reason for the observed decrease in permeate flux. Despite the high-temperature washing prior to the process, bacteria were still present in the deposits formed on the membranes (Figure 9). This confirms the conclusions of the paper [24] about the impossibility of completely eliminating biofouling by chemical washing. The largest decrease in permeate flux occurred after the installation was started up, after which the flux changes were at a similar level for the following days of DI water filtration. This may indicate that the contaminants generated during the filtration of real carwash wastewater were largely removed from the surface of the UF unit and that the observed flux decreases were mainly due to biofouling. Intensive bacterial development can be evidenced by the formation of numerous pilus/fimbria nanofibres in the deposits (Figure 9b).

### 3.6. Bacterial Analysis

The results shown in Figure 9 confirm that even the use of hot solutions for chemical washing did not completely eliminate the biofilm. The microbiological tests carried out showed that live bacteria were still present in samples taken from the membrane surface. One of the bacteria detected was *Bacillus cereus*, a Gram-positive bacterium. This bacterium produces spores that allow it to survive under very harsh conditions [29,30,32,44,45].

**Figure 10:**
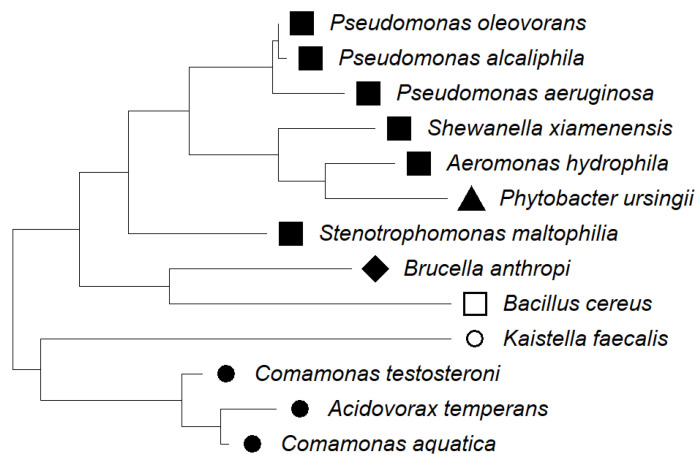
The phylogenetic tree of the bacteria identified on the membrane surface. Groups ♦ *Alphaproteobacteria*, ■ *Betaproteobacteria*, ● *Gammaproteobacteria*, ▲ *Enterobacteria*, □ *Bacillota*, ○ FCB group.

Thirteen species of bacteria were detected in the biofilm taken from the membrane surface, most of which belonged to the *Betaproteobacteria* group (Figure 10). 

The groups of microorganisms identified are consistent with those found in car wash effluent by other researchers [46,47]. Most of the bacteria identified are Gram-negative and rod-shaped, as confirmed by the shapes of the bacteria observed in the SEM studies (Figure 3, Figure 5, Figure 7 and Figure 9). Due to their distinctive structure, Gram-negative bacteria are more resistant than Gram-positive bacteria. Gram-negative bacteria have an envelope consisting of three layers, of which the performance of the outer membrane is the main reason for resistance to a wide range of chemicals and antibiotics [46]. In addition, Gram-negative bacteria have a high ability to form biofilms [39]. The formation of mixed-species biofilms further increases bacterial resistance to chemical compounds [48]. During membrane cleaning, the EPS produced by bacteria acts as a diffusion barrier, retarding convective flow and the transport of antimicrobial agents to microorganisms within the biofilm [25].

Among the bacteria detected were those that cause serious infections, such as *Phytobacter ursingii* and *Pseudomonas aeruginosa*, which belong to the *Enterobacteriaceae* group, and *Stenotrophomonas maltophilia*, which belongs to the *Betaproteobacteria* [46]. The formation of multi-species biofilms increased bacterial survival, making biofouling control more difficult [49,50].

### 3.7. Influence of Chemical Washing

**Figure 11:**
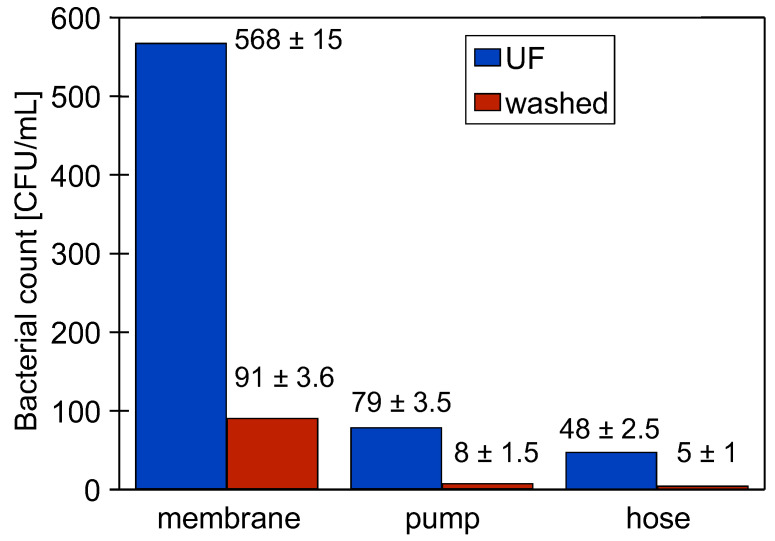
Effect of washing process application on the number of bacteria in the biofilm. UF–biofilm sample taken after the UF process (Figure 8).

After 5 days of DI water filtration (Figure 8), the effect of chemical washing on the reduction of bacteria in the biofilm formed during this test was investigated. The following wash programme was used: (a) 0.25% P3 Ultrasil 11 (323 K, 1 h), flush 2 L DI water, 0.5% H_3_PO_4_ (323 K, 1 h), flush 2 L DI water, 0.5% Insect (323 K, 1 h) and final flushing/rinsing with DI water. Before and after washing, biofilm samples were taken from the surface of the membranes (module B), from the inside of the hose (PVC) and from the pump inlet (acid-resistant steel) (Figure 2). The results presented in Figure 11 confirmed that a small number of bacteria survived long-term contact with the aggressive chemicals. Taking into account that the biofilm sample was dispersed in 10 mL of NaCl solution, it can be assumed that the number of bacteria per 1 cm^2^ of the surfaces examined was 10 times higher than that shown in Figure 11. The number of bacteria counted in this way on the surface of the pumps and hose after cleaning was similar to the number of bacteria (70–300 CFU/cm^2^) remaining on the surface of a dairy installation after a CIP procedure with agents containing NaOH and HNO_3_ [27].

Of the bacteria that survived the use of these agents, *Bacillus cereus* was the predominant bacteria found on the surface of acid-resistant steel. The use of superheated water (398 K for 30 min) allowed the *Bacillus* biofilm to be completely inactivated [28]. However, such a solution, used in the food industry, cannot be implemented in membrane modules, for which manufacturers usually specify 323 K as the limiting temperature.

After the UF process, there were five times more bacteria on the surface of the membranes than on the surface of the rest of the elements of the plant (Figure 11). The washing process drastically reduced the number of viable bacteria, but there were still significantly more bacteria on the membrane surface. Other work has shown that washing causes the surface biofilm to loosen, and the overflowing water detaches its fragments to form a planktonic form of biofilm [21,44]. It is also likely that in the UF plant studied, the flowing feed causes the biofilm to detach from the pipe surface. Their surface area was 687 cm^2^, which even at the low intensity of this phenomenon resulted in bacteria accumulating on the much smaller membrane surface (24 cm^2^).

**Figure 12:**
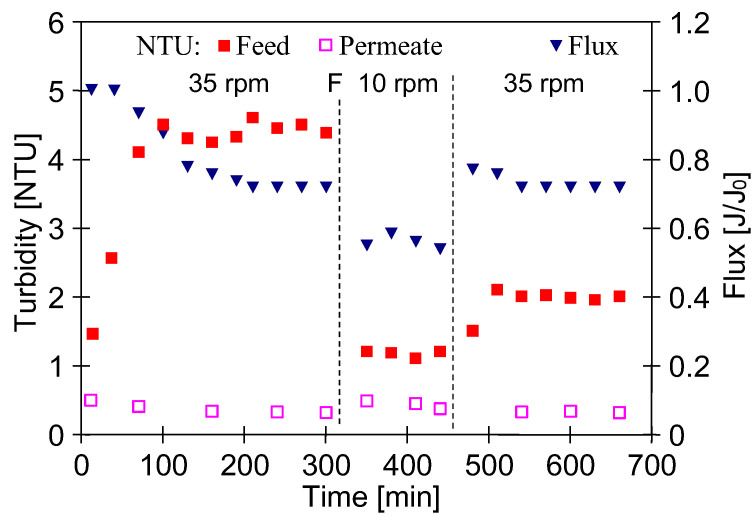
Effect of feed flow rate (rpm pump speed) on the permeate flux and changes in feed and permeate turbidity. Feed—DI water, point ‘F’—fresh portion of DI water. The dash lines−rpm values (division into periods).

To confirm the possibility of the formation of planktonic forms of biofilm after 5 days of plant standstill, new membranes were installed, and DI water filtration tests were carried out for different feed flow rates. The results, shown in Figure 12, confirmed that the feed flow rate had an effect on the changes in its turbidity, the only source of which could be the contaminants removed from the UF installation surface. The test was started with a pump speed of 35 rpm (1 m/s inside the modules). The increase in feed turbidity and the decrease in permeate flux indicate that suspension has appeared in the feed. After 315 minutes, the unit was flushed and filled with fresh DI water. To reduce the shear force, the test was started with a pump speed of 10 rpm, which was then increased to 35 rpm, resulting in an increase in feed turbidity. The lower flux value at 10 rpm confirms that the thickness of the deposit on the membrane surface can be reduced by increasing the feed flow rate [38].

**Figure 13:**
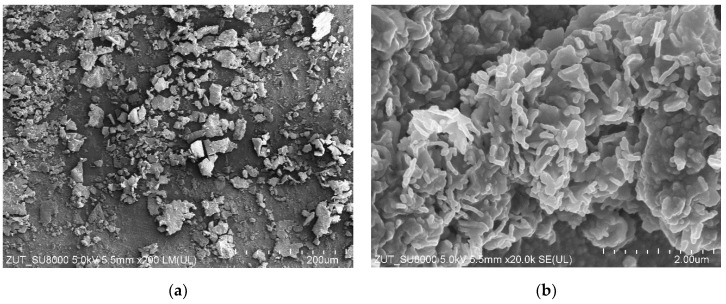
SEM images of seal surface: (**a**) deposits on seal, (**b**) rod-shaped bacteria in the deposits.

In addition to bacteria in the surface biofilm, bacteria that grow in areas of the system that are difficult for cleaning agents to penetrate can also cause secondary contamination of the system. One example is the rubber sealing rings on pump pistons. Despite their tight fit to the pump casing, SEM studies showed that the surface of the seals was covered with deposits (Figure 13a). Bacteria were also found in the deposit (Figure 13b), which could be a source of secondary contamination.

The cleaning agents used in membrane installations to remove biofilm are aggressive and can cause corrosion of plant components such as pumps [51]. The use of more corrosion-resistant components significantly increases the cost of the installation. Such costs are not acceptable to owners of small hand washes, which eliminates biofilm control by most of the commonly used chemical agents. However, daily rinsing of the UF system with alkaline agents, such as Insect solutions used in car washes, has been shown to greatly reduce the development of biofouling [17].

## 4. Conclusions

Bacteria present in car wash wastewater during the UF process form a biofilm layer not only on the membrane surface, but also on the surface of all plant components.

Despite repeated chemical cleaning of the UF installation with H_3_PO_4_ acid and alkaline detergents containing NaOH over many hours, it was not possible to remove the biofilm from the elements of the UF installation. As a result, during the DI water filtration, bacteria detached from the pipe surface and caused biofouling of the membranes. For this reason, not only periodic module washing but also intensive chemical cleaning of UF unit components should be carried out.

As a result of biofouling, the permeate flux decreased by a factor of two after only 10 h of UF process using DI water as feed. Washing with hot alkaline solutions such as P3 Ultrasil 11 (pH = 12) significantly increased the performance of the UF modules but did not restore the initial permeate flux.

As a result of chemical washing, the number of viable bacteria in the biofilm decreased by a factor of 10, to levels of 50–100 CFU/cm^2^ on the surface of the UF unit and close to 1000 CFU/cm^2^ on the surface of the membranes. These bacteria in the biofilm, as well as bacteria surviving in hard-to-reach areas of the plant, such as pump seals, can be a source of secondary bacterial growth in the UF plant.

After repeated chemical washes, it was found that in addition to *Bacillus cereus* bacteria, several types of Gram-negative bacteria, which are highly resistant to chemicals, survived in the UF system. Therefore, in the case of UF car wash effluent, the use of periodic washing will only reduce the amount of biofouling and will not eliminate it.

## Figures and Tables

**Table 1:** Composition of alkaline cleaning agents.

Cleaning Agent	Component	Concentration [wt.%]
	NaOH	>40
P3 Ultrasil 11	ethylenediaminetetraacetic acid tetrasodium salt	>30
(powder)	anionic surfactants	<5
	non-ionic surfactants	<5
	NaOH	3–5
Insect	ethylenediaminetetraacetic acid tetrasodium salt	3–5
(solution)	diethylene glycol butyl ether	3–5
	sulfonic acids, C14-16-alkane hydroxy and C14-16-alkene, sodium salts	5–7

**Table 2:** SEM-EDX analysis of deposits formed on the UE50 membrane (Figure 7).

Element	After UF	After Washing
C	47.67	43.59
O	42.34	42.66
S	6.92	12.55
Si	2.07	0.99
P	0.29	0.14
Mg	0.12	−
Ca	0.17	0.07
Fe	0.42	−

## Data Availability

The original contributions presented in the study are included in the article. The related raw data generated during the study (2024) were deposited by the authors of this article in the Most Wiedzy repository and are available from: https://mostwiedzy.pl/pl/open-research-data/separation-of-bacteria-in-uf-process,1004053202102190-0 (accessed on 15 December 2024).
